# Physical Exercise and Sleep Quality Among Chinese College Students: The Mediating Role of Self-Control and the Moderating Role of Mindfulness

**DOI:** 10.3390/bs15020232

**Published:** 2025-02-18

**Authors:** Xiaopeng Li, Chengli Xu, Wanyi Chen, Jing Tian

**Affiliations:** 1 School of Physical Education and Sports, Central China Normal University, Wuhan 430079, China; xiaoepngli@mails.ccnu.edu.cn (X.L.); xuchengli007@163.com (C.X.); taratianjing@ccnu.edu.cn (J.T.); 2 School of Psychology, South China Normal University, Guangzhou 510631, China

**Keywords:** physical exercise, sleep quality, self-control, mindfulness

## Abstract

Although a few studies have examined the relationship between physical exercise and sleep quality, the underlying mechanisms of this association remain unclear. This study aims to investigate how and under what conditions physical exercise can promote the sleep quality among Chinese college students, with a focus on how self-control mediates and mindfulness moderates this relationship. Data were collected through convenient sampling from five universities in China, with a total of 1028 college students aged 16 to 29 participating in the study. Participants were recruited to complete the self-report questionnaires measuring their physical exercise, self-control, sleep quality and mindfulness. Results showed that physical exercise significantly and positively predicts sleep quality among Chinese college students. Further moderated mediation analyses indicated that self-control plays a significant mediating role, while mindfulness plays a moderating role in this relationship. Additionally, simple slopes analyses found that the moderating effect of mindfulness is more pronounced among individuals with lower levels of mindfulness compared to those with higher levels. The findings suggest that physical exercise can enhance sleep quality by improving self-control. Furthermore, physical exercise has a beneficial impact on self-control and sleep quality among college students with low mindfulness, while mindfulness itself exerts a distinct positive influence among those with high mindfulness.

## 1. Introduction

According to a survey conducted by the World Health Organization, a quarter of adolescents have been reported feeling nervous, irritable or experiencing sleep difficulties each week (World Health Organization, 2021), and sleep disorders have emerged as a significant threat to public health worldwide (Hale et al., 2020). Notably, insomnia is particularly prevalent among college students, with an incidence rate as high as 18.5%, which is much higher than that of other age groups (Koyanagi & Stickley, 2015). Currently, poor sleep quality is a particularly serious problem among Chinese college students (Wu et al., 2021). A survey conducted in China indicates that 25.7% of Chinese college students experience sleep problems, a figure far exceeding the 7.4–15.0% reported in the general population (Q. Q. Liu et al., 2018). Therefore, numerous initiatives aimed at enhancing the sleep quality of Chinese college students have been proposed. In this context, researchers have been widely concerned with how physical exercise affects the sleep quality of college students.

In addition, as research in positive psychology has been progressively deepened, certain positive protective factors, including self-control (J. Liu et al., 2020) and mindfulness (Lentz & Brown, 2019), have garnered increasing attention. Previous studies have found that self-control plays a significant mediating role between physical exercise and health problems such as sedentary behavior (Boat et al., 2024), and a strong link exists between sedentary behavior and sleep quality among Chinese college students (X. Zhang et al., 2022), meaning that self-control likely serves as a mediator in the relationship between physical exercise and sleep quality among college students. Therefore, we introduced self-control to further examine whether it mediates the relationship between physical exercise and sleep quality. In addition, previous studies have proven that mindfulness is a strong enhancer that can enhance the positive impact of physical exercise on a healthy lifestyle (Ruffault et al., 2016), and mindfulness can play a moderating role between healthy lifestyle and sleep quality in college students (L. Zhang et al., 2022). This suggests, from an alternative theoretical perspective, that mindfulness might serve as a regulatory factor in the process by which physical exercise influences the sleep quality of college students. Therefore, the present study introduces mindfulness to explore its moderating role in the above mediating model. Although the positive relationship between physical exercise and sleep quality has been proved (Mahfouz et al., 2020), research on the potential mediating and moderating mechanisms of this relationship is still limited. It is necessary to explore the influencing factors and potential influence mechanisms of physical exercise on the sleep quality of college students. Therefore, this study constructed a moderated mediation model to explore the underlying mechanisms between physical exercise and sleep quality.

### 1.1. Physical Exercise and Sleep Quality

Physical exercise is a physical activity with a certain intensity, frequency, and du-ration to improve health (Jiang et al., 2009). On the one hand, studies have shown that regular physical exercise is a positive and non-drug treatment for those with poor sleep quality. Specifically, physical exercise can improve the negative psychological emotions of college students, such as depression (Slykerman et al., 2020), anxiety (Patterson et al., 2021), and stress (Herawati & Gayatri, 2019), which could further improve their sleep quality. On the other hand, experimental studies have also shown that regular physical exercise in individuals can increase melatonin secretion levels in individuals, which can enhance their sleep quality (Chennaoui et al., 2015). All these benefits of physical exercise mentioned above can help to improve college students’ sleep quality. In consideration of the above, we proposed the following hypothesis:

**Hypothesis** **1.** *Physical exercise may have a positive impact on sleep quality among Chinese college students.*

### 1.2. The Mediating Role of Self-Control

Self-control is the ability of an individual to regulate reactions to overcome impulses, which requires individuals to independently adjust their behavior and conform to social expectations and their own values by changing their own behavior, way of thinking, and level of consciousness (MacLean et al., 2014). It has been verified that an individual’s self-control is consistently and closely related to health behaviors. Specifically, an individual’s self-control is positively related to health-promoting behaviors and negatively related to health-damaging behaviors (Hagger et al., 2019a). Poor sleep quality among college students due to sleep latency is an unhealthy behavior that implies a failure of individual self-control (Kroese et al., 2016). This means that college students succumb to other temptations before going to sleep, resulting in delayed sleep and poor sleep quality. Based on the fact that self-control is a limited resource, relevant studies further show that college students expend a lot of self-control resources during the day, resulting in the least self-control resources at night. This means there is a greater tendency for sleep procrastination in individuals with relatively depleted self-control resources, which can lead to poorer sleep quality (Bernecker & Job, 2020).

Physical exercise has been shown to be an effective way to improve individual self-control. Specifically, individuals who participate in regular physical exercise tend to have higher levels of self-control, which in turn affects the management of individuals’ resources to implement goal-directed behaviors to achieve long-term goals, such as developing healthy sleep habits (Eysenck et al., 2007). Interestingly, it has been reported that self-control can play a mediating role between physical exercise and healthy lifestyle habits in college students (Hagger et al., 2019b), and an experimental study has been also observed that aerobic exercise may be a potentially effective intervention to enhance self-control in female college students, which could be beneficial in helping college students to develop their healthy habits (Chen et al., 2019). Sleep quality is an important organizing component of healthy lifestyle habits. Although there is no direct evidence that self-control can directly mediate the relationship between physical exercise and sleep quality, considering the similarities between sleep quality and healthy lifestyle habits, we hypothesized that self-control may play a mediating role in the relationship between physical exercise and sleep quality among college students. Thus, we proposed the following hypothesis:

**Hypothesis** **2.** *Self-control may act as a mediator in the link between physical exercise and sleep quality among college students.*

### 1.3. The Moderating Role of Mindfulness

Mindfulness refers to being aware of the present reality or current experience in an accepting or non-judgmental way (Brown & Ryan, 2003). Previous research has shown that high mindfulness could promote physical and mental health such as sleep quality, positive effects, and self-control (Bajaj et al., 2016; Lyddy et al., 2021). In recent years, a growing number of studies have confirmed that high levels of mindfulness can play an enhancing or buffering role in self-control and sleep quality in college students, such as a study by Lentz and Brown (Lentz & Brown, 2019), who suggested that the relationship between physical exercise and sleep quality was intensified for college students with high mindfulness.

According to the re-perceiving model of mindfulness, mindfulness can help people re-perceive the moment-by-moment experience with greater objectivity, get rid of automatic behavioral and emotional patterns, and facilitate adaptive responses to negative stimulation (Shapiro et al., 2006), which means that mindfulness can buffer the adverse effects of negative factors on an individual. Indeed, it is also true that previous studies have confirmed that mindfulness can buffer the adverse effects of negativity, such as in Davis et al. (2016), who showed that mindfulness can relieve college students’ anxiety, depression, and other negative psychological emotions, which has been proven to be one of the important factors for promoting the ability of college students to improve their sleep quality. In other words, mindfulness acts as a buffer with respect to psychological distress for individuals, which can help people avoid sleep disorders and improve sleep quality on a psychological level. Furthermore, according to the protection–protection model, two different protective factors will interact with each other, increasing or decreasing the outcome (Boksem et al., 2006). Specifically, the enhancing interaction hypothesis suggests that the effect of one protective factor (e.g., physical exercise) on its outcome (e.g., self-control) may be enhanced by another protective factor (e.g., mindfulness). Namely, individuals with high levels of mindfulness showed stronger associations between physical exercise and self-control than individuals with low levels of mindfulness. For example, a study indicated that the relationships between physical exercise and self-control were moderated by mindfulness, in that they were stronger for adolescents with higher levels of mindfulness (Stocker et al., 2019). Another study indicated that the direct effect of deprivation on sleep quality and its indirect effect through social anxiety were both moderated by mindfulness, in that those two effects were weaker in individuals with high levels of mindfulness (Xiong et al., 2023). Thus, mindfulness serves as an important protective factor that can reinforce the positive effects of physical exercise. In other words, the link between physical exercise and sleep quality, as well as the link between physical exercise and self-control, may be moderated by mindfulness. Above all, we propose the hypothesis as follows:

**Hypothesis** **3.** *Mindfulness would moderate the relationship between physical exercise and sleep quality as well as physical exercise and self-control.*

### 1.4. Research Objective

**Figure 1:**
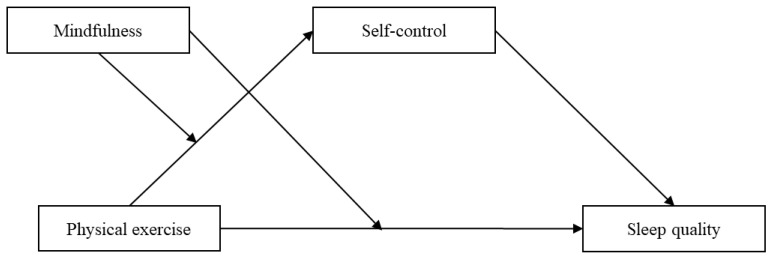
The proposed moderated mediation model.

Although the precise mechanisms remain not fully elucidated, substantial evidence suggests significant associations between physical exercise, sleep quality, self-control, and mindfulness. We posit that integrating these factors into a comprehensive model could effectively broaden the research perspective on sleep quality among college students. Taken together, we constructed a moderated mediation model in this study (Figure 1).

The objective of our study was to investigate the impact of physical exercise on sleep quality among college students, while examining the mediating role of self-control and the moderating role of mindfulness in this relationship. Our findings aim to uncover the underlying mechanisms contributing to poor sleep quality among college students and to offer solutions to enhance sleep quality among Chinese college students.

## 2. Methods

### 2.1. Participants

A total of 1141 students participated in our study. After excluding participants who provided incomplete information and those who completed the survey in an unrealistically short time, the final sample consisted of 1028 participants. Among them, there were 487 (47.37%) males and 541 (52.63%) females. Their average age was 19.14 years old (*SD* = 1.85), ranging from 16 to 29 years old. Among the participants, 399 were freshmen (38.79%), 395 were sophomores (38.45%), 105 were juniors (10.17%), and 129 were seniors (12.76%). Additionally, there were 19 first-year graduate students (1.90%), 32 second-year graduate students (3.10%), and 50 third-year graduate students (4.83%).

### 2.2. Procedure

This study was conducted in accordance with the principles of the Declaration of Helsinki (World Medical Association, 2013) and approved by the Ethics Committee for Scientific Research at the authors’ institution. Data were collected through convenience sampling from 5 comprehensive universities in Hubei Province, a major province in central China. The survey was conducted through an online platform called Wen Juan Xing (https://www.wjx.cn/) from 24 May to 21 June 2024. Prior to the study, all participants received an electronic informed consent, which provided detailed information about the survey and its procedure. Participants were also informed that they could terminate their participation at any time without any repercussions. Students who agreed to participate then completed the surveys by scanning the Quick Response (QR) code of the questionnaire at their school using their mobile phones or computers. It took approximately 10 min for all participants to complete the anonymous self-reported questionnaires.

## 3. Measures

### 3.1. Physical Exercise

Physical exercise was assessed using the Physical Activity Rating Scale (PARS-3), developed by Liang (1994). This scale consists of three items measuring the exercise intensity, exercise time, and exercise frequency of the participants to assess their physical activity level. Exercise frequency, exercise time, and exercise intensity were scored from 1–5. The physical activity level of the test subjects was calculated according to the formula of physical activity amount = exercise intensity × (exercise time − 1) × exercise frequency. The higher the score measured by the scale, the higher the level of physical activity of the participants. Cronbach’s alpha coefficient for the PARS-3 was 0.79.

### 3.2. Sleep Quality

This study used the Chinese version of the Pittsburgh Sleep Quality Index (PSQI, X. Liu, 1996), which was adapted from the original scale developed by Buysse et al. (1989). Previous studies have shown that the Chinese version of the PSQI has demonstrated good reliability and validity (Zheng et al., 2016). The PSQI consists of seven dimensions, including 19 items measuring individuals’ subjective sleep quality, sleep latency, sleep duration, habitual sleep efficiency, sleep disturbance, sleep medication used, and daytime dysfunction. All items were scored from 0–3 and the total score ranged from 0–21. In the original scale, higher scores indicate poorer sleep quality. For better understanding, we have reversed all the items in this study. The higher the score, the better the individual’s sleep quality. In this study, Cronbach’s alpha for the PSQI was 0.82.

### 3.3. Self-Control

The Chinese version of the Self-Control Scale was used in our study (Tan & Guo, 2008). This scale comprises five dimensions, including 19 items that measure individuals’ self-control through resisting temptation, impulse control, healthy habits, task performance, and entertainment temperance. Participants responded to the 19 items on a five-point Likert-type scale ranging from 1 (not at all) to 5 (very much), with a higher total score indicating better self-control. In the current study, the items demonstrated acceptable reliability (Cronbach’s α = 0.82).

### 3.4. Mindfulness

In this study, we used the Child and Adolescent Mindfulness Measure (CAMM, Greco et al., 2011) to measure the participants’ level of mindfulness. The CAMM consists of 10 items. Participants rated their agreement with these items on a five-point Likert scale, ranging from 0 (never) to 4 (always). All responses were averaged after reversing the reverse-scored item. The higher the score, the higher the level of mindfulness in daily life. In this study, Cronbach’s alpha of the CAMM was 0.81.

### 3.5. Control Variables

Gender and age were included as control variables in our study because previous studies found that they were closely associated with the main variables in this study (Lueke & Assar, 2024; Li et al., 2016).

### 3.6. Data Analysis

Data management and analysis were performed using SPSS 29.0 and the PROCESS macro developed by Hayes in 2013 (Version 3.5; https://www.processmacro.org/download.html, accessed on 1 November 2024). First, missing data accounted for less than 5% and were handled using the expectation maximization (EM) approach before conducting subsequent analyses (Schlomer et al., 2010). Next, a Harman single factor test was conducted to address potential common method biases (Podsakoff et al., 2003). Third, descriptive analysis of the main variables and Pearson correlation analysis were performed. Considering that data standardization in moderated mediation analysis helps to eliminate scale differences, reduce multicollinearity, and enhance model interpretability and stability (Hayes & Rockwood, 2016), we standardized the data.

Fourth, we conducted a Bootstrap analysis using the SPSS PROCESS macro (model 8) suggested by Hayes to test the proposed moderated mediation model. In addition, simple slopes analyses were performed to test all the potential significant interaction effects. Bootstrap confidence intervals (95% CIs) based on 5000 random samples of the data were utilized to determine the significance of the regression coefficients in Model 8. The effects were considered significant if the 95% CI did not include a zero value (Preacher et al., 2007). In addition, simple slopes analyses were conducted to examine all potential significant interaction effects. The simple slope test, also known as the pick-a-point approach or spotlight analysis (Hayes, 2017), is a conditional hypothesis test that assesses whether the relationship between an independent variable and a dependent variable is significant at specific values of the moderator (Hayes, 2015; Memon et al., 2019). In our analysis, we used the ±1 standard deviation method to probe the effects of mindfulness at different levels. This approach allows us to evaluate the moderating effects of mindfulness without altering its continuous nature, thereby providing a detailed understanding of how mindfulness influences the relationships between physical exercise and sleep quality, as well as physical exercise and self-control.

## 4. Results

### 4.1. Analysis of Common Method Bias

Considering that this study adopts the method of the questionnaire survey, there may be the risk of common method bias (Tehseen et al., 2017). Although this study took some measures to reduce the common method bias in the process of data distribution, such as selecting a measurement instrument with high reliability and validity, anonymous responding, and using reverse scoring for scale items, the common method bias may still exist. Thus, we tested it by using the Harman single-factor method (Podsakoff et al., 2003). The results showed that there were 10 factors with eigenvalues greater than 1, among which the variation explained by the first factor was 14.79%, which was less than the critical criterion of 40%. Therefore, it could be concluded that there was no significant common method bias in the data of this study.

### 4.2. Preliminary Analyses

The means (*M*), standard deviations (*SD*), and correlations for all of the observed variables are presented in Table 1. As hypothesized, physical exercise was positively correlated with both self-control (*r* = 0.32, *p* < 0.001) and sleep quality (*r* = 0.26, *p* < 0.001). Self-control was positively correlated with both sleep quality (*r* = 0.40, *p* < 0.001) and mindfulness (*r* = 0.29, *p* < 0.001). Mindfulness was positively correlated with sleep quality (*r* = 0.51, *p* < 0.001).

### 4.3. Testing for the Proposed Moderated Mediation Model

**Figure 2:**
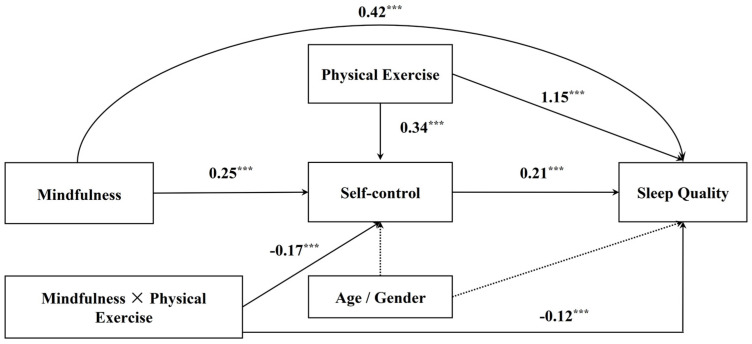
Results for the hypothesized moderated mediation model. Note: *N* = 1028. *** *p* < 0.001.

In this study, we adopted Hayes’s (2013) PROCESS macro (Model 8) to examine the proposed moderated mediation model. The main results of the moderated mediation analysis were shown in Figure 2 and Table 2.

After controlling for gender and age, moderated mediation analyses were conducted. As expected, physical exercise showed a positive and significant total effect on sleep quality, with a regression coefficient being 0.26 (95% CI [0.19, 0.32], *p* < 0.001). When self-control was included as a mediator in the regression equation, physical exercise could still significantly predict sleep quality (β = 0.13, 95% CI [0.07, 0.19], *p* < 0.001). Moreover, self-control showed a positive and significant effect on sleep quality (β = 0.36, 95% CI [0.30, 0.42], *p* < 0.001). Physical exercise also significantly predicted self-control (β = 0.35, 95% CI [0.28, 0.41], *p* < 0.001). Furthermore, a Sobel test was employed to examine the significance of the indirect effect of self-control. Results showed that self-control significantly mediated the relationship between physical exercise and sleep quality (*Z* = −8.08, *p* < 0.001). These results provided compelling evidence that physical exercise can improve the sleep quality of college students, and this relationship is mediated by self-control. Thus, Hypotheses 1 and 2 were supported.

The solid line represents a significant effect and the dashed line represents a non-significant effect.

To test Hypothesis 3, mindfulness was included as a moderator in the regression equation. These results showed that mindfulness could positively and significantly predict self-control (β = 0.25, 95% CI [0.19, 0.30], *p* < 0.001) and sleep quality (β = 0.42, 95% CI [0.37, 0.48], *p* < 0.001). Furthermore, two interaction effects were further analyzed. The results showed that there was a significant physical exercise × mindfulness effect on self-control (β = −0.17, 95% CI [−0.22, −0.12], *p* < 0.001) in the mediator variable model. Furthermore, the effect of physical exercise × mindfulness on sleep quality (β = −0.12, 95% CI [−0.17, −0.07], *p* < 0.001) was also significant in the dependent variable model. These results suggest that both the relationship between exercise and sleep quality and the relationship between exercise and self-control were moderated by mindfulness. Hypothesis 3 was supported.

**Figure 3:**
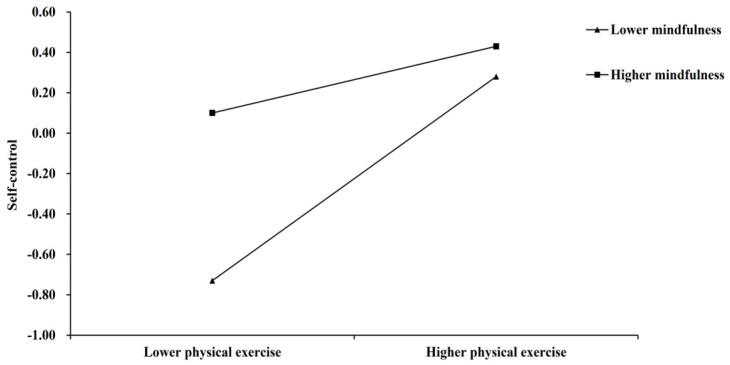
Mindfulness moderates the relationship between physical exercise and self-control.

**Figure 4:**
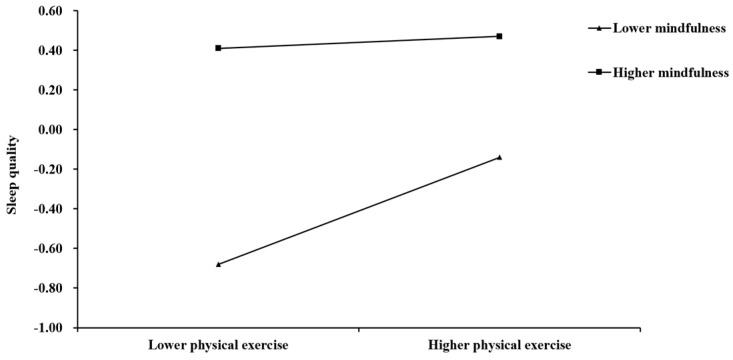
Mindfulness moderates the relationship between physical exercise and sleep quality.

Additionally, simple slope analyses were conducted to further explain the moderating effect. Specifically, these analyses investigated whether the slopes for individuals with high mindfulness capacity (one standard deviation above the mean) differed from those of individuals with low mindfulness capacity (one standard deviation below the mean) in both mediator and dependent variable models. For visual inspection of the direction and strength of the moderating effect, we report the simple slope plots (see Figure 3 and Figure 4). As shown in Figure 3, physical exercise could positively and significantly predict self-control for college students with both high and low mindfulness. Interestingly, the effect of physical exercise on self-control was stronger for those with lower mindfulness (β = 0.51, *t* = 12.23, 95% CI [0.42, 0.58], *p* < 0.001) than for those with higher mindfulness (β = 0.17, *t* = 4.28, 95% CI [0.06, 0.24], *p* < 0.001). Furthermore, as shown in Figure 4, the effect of physical exercise on sleep quality was positive and significant for college students with low mindfulness (β = 0.27, *t* = 6.77, 95% CI [0.19, 0.35], *p* < 0.001), whereas it was not significant for those with high mindfulness (β = 0.03, *t* = 0.88, 95% CI [−0.04, 0.10], *p* > 0.05). These findings indicate that physical exercise has a beneficial impact on self-control and sleep quality among individuals with low mindfulness, while mindfulness itself exerts a distinct influence among those with high mindfulness. Specifically, for individuals with low mindfulness, physical exercise significantly enhances both sleep quality and self-control. In contrast, for individuals with higher levels of mindfulness, although physical exercise still promoted self-control, its effect on sleep quality was no longer significant.

## 5. Discussion

### 5.1. Physical Exercise and Sleep Quality

The present study demonstrates that physical exercise significantly and positively correlates with the sleep quality of college students. These findings are consistent with prior research investigating the impact of physical activity on sleep quality among college students (Bodziony & Stetson, 2024). Thus, Hypothesis 1 has been confirmed. However, our study further suggests that multiple factors may underlie the positive association between physical exercise and sleep quality among college students, with both physiological and psychological aspects providing explanatory frameworks for these results. From a physiological perspective, numerous studies have established a clear connection between body temperature regulation and sleep quality (Chang et al., 2009; Quanten et al., 2006). For example, physical exercise triggers an elevation in central, skin, and cerebral temperatures. Consequently, under the thermoregulatory mechanism, peripheral blood vessels dilate, facilitating heat emission to the periphery and resulting in a decrease in overall body temperature. Research indicates that a reduction in body temperature by 0.5–1 °C enhances the likelihood of falling asleep and promotes better sleep quality Moreover, post-exercise changes in body surface temperature, particularly the gradient between distal and proximal skin temperatures, may serve as a pivotal factor in initiating sleep onset for individuals (Kräuchi, 2007). Additionally, exercise induces alterations in serotonin, epinephrine, melatonin, and cortisol levels, all of which have been linked to variations in sleep quality (Chennaoui et al., 2015). From a psychological point of view, improving college students’ emotional state through exercise has been shown to be an important additional factor in improving their sleep quality (Ghrouz et al., 2019). Specifically, many adverse psychological emotions such as anxiety, depression (Slykerman et al., 2020), and stress (Herawati & Gayatri, 2019) could affect the sleep quality of college students. Further studies have shown that improving the above-mentioned psychological emotions can enhance the sleep quality of college students (Johnston et al., 2021).

In summary, our study shows that, on the one hand, proper physical activity can help college students fall asleep faster and have a better sleep quality. Several physio-logical factors such as hormones, fatigue, and body temperature changes may provide an explanation for this phenomenon. On the other hand, engaging in appropriate physical activity can also alleviate the psychological distress of college students, which can im-prove their sleep quality from a psychological perspective.

### 5.2. The Mediating Role of Self-Control

Our study elucidates the mediating role of self-control in influencing the relationship between physical exercise and sleep quality among college students. In line with previous research, self-control has been identified as a mediator in the association between physical exercise and the adoption of healthy lifestyle habits (Kuang et al., 2023). Our investigation extends this understanding by demonstrating that self-control significantly moderates the relationship between physical exercise and sleep quality in college students. Specifically, Chinese college students who regularly participate in physical exercise exhibit higher levels of self-control, leading to enhanced sleep quality. As illustrated in the first half of this study, according to the self-control resource model, when college students conduct self-control activities during the day and consume part of their self-control resources, their self-control resources are often deficient at night. At this time, college students do not easily resist temptation (such as using mobile phones, etc.), which can disrupt sleep patterns and reduce sleep quality. However, it has been confirmed that physical exercise could improve the self-control ability of college students; that is, college students who often participate in physical exercise often have a higher level of self-control ability and more self-control resources, which makes it easier for them to resist various external temptations and improve their sleep quality. In other words, adequate sleep can improve individuals’ self-control ability, and when they have more self-control resources, they are more likely to resist temptation, make sleep a higher priority, and have better-quality sleep. Our study further suggests that, through physical exercise, improved self-control ability could improve the sleep quality of college students, which may serve as a theoretical guideline for future family education and school education in improving the sleep quality of college students.

### 5.3. The Moderating Role of Mindfulness

One significant finding from our study was the potential moderating effect of mindfulness on the relationship between physical exercise and self-control. Specifically, both the direct effect that physical exercise itself exerted on sleep quality and the indirect effect of self-control were moderated and buffered by mindfulness, with these effects being stronger for college students with lower levels of mindfulness. This outcome suggests that mindfulness, as a positive personality trait, may enhance the self-control capacities of college students during physical exercise. As their self-control strengthens, they are better equipped to resist negative temptations, thereby potentially improving their sleep quality. This finding is consistent with the enhancing interaction hypothesis in the protective model (Wills et al., 1992), which posits that both mindfulness and self-control serve as protective factors, with each factor enhancing the effects of the other. Specifically, self-control is recognized as a protective factor that provides individuals with the executive function necessary to avoid staying up late, resist poor optimization, and pursue a healthy lifestyle. Mindfulness is also regarded as another protective factor that enhances individuals’ awareness of the health risks associated with staying up late and experiencing poor sleep quality, thereby facilitating these processes. That is, individuals with higher levels of mindfulness exhibit lower sensitivity compared to those with diminished levels of mindfulness in the context of self-control’s impact on sleep quality. The findings from our study reveal an intriguing result: the simple slope at low levels of mindfulness was significantly greater than that at high levels of mindfulness. This suggests that for college students exhibiting low levels of mindfulness, physical exercise serves as a highly effective means to enhance self-control, with mindfulness functioning primarily as a complementary factor in this process. This finding presented above offers a novel perspective in terms of enhancing the sleep quality of college students: it is essential to focus more on college students with low levels of mindfulness than those with high levels.

Furthermore, this finding also further supports the Metacognitive Model of Insomnia, which model contends that an individual’s ability to accurately observe internal and external experiences (e.g., mindfulness) and respond appropriately to external stimuli related to sleep significantly influence sleep quality (Ong et al., 2012). This suggests that college students with higher mindfulness are more attuned to both their internal and external experiences, thereby gaining a clearer appreciation of the health benefits associated with improved sleep quality. Consequently, individuals with higher mindfulness levels tend to exhibit greater adaptability in addressing sleep disorders, often resorting to positive coping mechanisms, potentially enhancing their sleep quality. The aforementioned phenomenon further elucidates why individuals with high levels of mindfulness do not show strong sensitivity to the effects of physical exercise on sleep quality. It is particularly intriguing to observe from the simple slope graph that as levels of mindfulness increase, the impact of physical exercise on sleep quality diminishes. Notably, we encountered a counterintuitive finding indicating that physical activity did not significantly influence sleep quality in individuals with higher levels of mindfulness. This observation further underscores that the relationship between physical exercise and sleep quality among col-lege students constitutes a holistic and systematic process; thus, understanding this in-ternal dynamic cannot be fully achieved by examining each component in isolation. For instance, our results indicate that when analyzing the effects of physical exercise and mindfulness on college students’ sleep quality separately, both factors exhibit positive influences. However, when exploring their interaction concerning sleep quality, we encounter inconsistent outcomes. This suggests that in both research and practical ap-plications, it is essential to avoid viewing the effects of physical exercise and sleep quality in isolation. Instead, considering their interplay is crucial for comprehensively and accurately addressing issues related to sleep quality. Thus, it is imperative for relevant authorities, such as educators and practitioners, to recognize an intriguing insight: individuals with higher levels of mindfulness were significantly less likely to have sleep risks, and encouraging them to participate in physical exercise did not improve their sleep quality. On the contrary, for individuals who do not possess a strong inclination towards physical activity, enhancing their level of mindfulness appears to be an effective strategy for improving sleep quality.

### 5.4. Limitations and Future Directions

Although our study contributes new evidence regarding the mediating role of self-control and the moderating effect of mindfulness on the relationship between physical exercise and sleep quality in college students, it is essential to recognize several limitations. Firstly, our study utilized a cross-sectional design, which precludes establishing definitive causal relationships between variables. Secondly, the use of convenience sampling and the restriction to participants from only Hubei Province may introduce sampling biases and limit the generalizability of our findings. Additionally, our investigation focused solely on two internal factors, namely self-control and mindfulness, without considering a broader array of external influences.

To address the limitations of our study and further explore the impact of physical exercise on college students’ sleep quality, future research could be enhanced in the following ways: Firstly, given the methodological limitations, subsequent studies should employ longitudinal follow-up research to ascertain the causal relationships between variables more accurately. Secondly, considering the limitations of our sampling methods and participant selection, future research endeavors could broaden the dataset and explore the model using random or stratified sampling techniques to examine diverse and larger cohorts. Lastly, acknowledging the constraints of our current understanding of influencing mechanisms, future research could incorporate additional variables, particularly external factors such as family environment (Counts et al., 2018) and school support (Wang & Bíró, 2021). This broader scope of investigation would enable a more comprehensive exploration of how these external factors interact to influence college students’ sleep quality.

## 6. Conclusions

The results of this study reveal a significant positive correlation between physical exercise and the sleep quality of college students, which is mediated by self-control and moderated by mindfulness. Specifically, physical exercise can not only directly improve the sleep quality of college students, but also enhance their sleep quality indirectly through self-control. In addition, mindfulness plays a crucial moderating role in both the direct and indirect pathways mentioned above. Notably, the moderating effect of mindfulness is more pronounced among individuals with lower levels of mindfulness compared to those with relatively higher mindfulness levels. Physical exercise has a beneficial impact on self-control and sleep quality among college students with low mindfulness, while mindfulness itself exerts a distinct influence among those with high mindfulness. Overall, while our study acknowledges certain limitations, it significantly contributes to the understanding of the interplay between physical exercise, sleep quality, self-control, and mindfulness among college students. Furthermore, this research offers valuable insights for parents and educators in developing programs aimed at promoting sleep quality and fostering healthy living policies for adolescents.

## Figures and Tables

**Table 1:** Descriptive statistics and interrelations among all of the observed variables.

Variables	*M*	*SD*	1	2	3	4	5	6
1. Gender ^a^	1.53	0.50	1					
2. Age	19.14	1.85	−0.03	1				
3. Physical exercise	2.48	1.03	−0.34 ***	0.22 ***	1			
4. Self-control	3.30	0.63	−0.07 *	0.23	0.32 ***	1		
5. Sleep quality	−4.93	2.9	−0.11 ***	0.02	0.26 ***	0.40 ***	1	
6. Mindfulness	2.47	0.72	−0.10 **	0.06	0.13 ***	0.29 ***	0.51 ***	1

Note. *N* = 1028. *** *p* < 0.001, ** *p* < 0.01, * *p* < 0.05. ^a^ 0 = male, 1 = female.

**Table 2:** Regression results for the conditional indirect effect (moderated mediation).

Model									
Model 1: Total effect model (outcome variable: sleep quality)									
*R*	*R* ^2^	*F*	*df*_1_	*df*_2_	*p*	β	*SE*	*t*	*p*
0.26	0.07	24.97	3	1024	<0.001				
Constant						5.41 ***	0.97	5.59	<0.001
Gender						−0.03	0.03	−0.84	0.40
Age						−0.04	0.03	−1.15	0.25
Physical exercise						0.26 ***	0.03	7.80	<0.001
Model 2: Mediator variable model (outcome variable: self-control)									
*R*	*R* ^2^	*F*	*df*_1_	*df*_2_	*p*	β	*SE*	*t*	*p*
0.45	0.21	53.25	5	1022	<0.001				
Constant						1.61 **	0.24	6.60	<0.001
Gender						0.07	0.04	1.84	0.06
Age						−0.02	0.01	−1.81	0.07
Physical exercise						0.34 ***	0.03	11.03	<0.001
Mindfulness						0.25 ***	0.03	8.69	<0.001
Physical exercise × Mindfulness						−0.17 ***	0.03	−6.48	<0.001
Model 3: Dependent variable model (outcome variable: sleep quality)									
*R*	*R* ^2^	*F*	*df*_1_	*df*_2_	*p*	β	*SE*	*t*	*p*
0.6	0.36	97.18	6	1021	<0.001				
Constant						0.01	0.03	0.60	0.55
Gender						−0.12	0.27	−0.68	0.50
Age						−0.04	0.03	−1.42	0.16
Physical exercise						1.15 ***	0.03	5.19	<0.001
Self-control						0.21 ***	0.03	7.47	<0.001
Mindfulness						0.42 ***	0.03	16.26	<0.001
Physical exercise × Mindfulness						−0.12 ***	0.02	−4.96	<0.001
Conditional direct effect analysis at values of mindfulness (*M* ± *SD*)									
						β	Boot SE	LLCI	ULCI
*M* − 1*SD* (1.75)						0.27 ***	0.04	0.19	0.35
*M* (2.47)						0.15 ***	0.03	0.09	0.21
*M* + 1*SD* (3.22)						0.03	0.04	−0.04	0.10
Conditional indirect effect analysis at values of mindfulness (*M* ± *SD*)									
						β	Boot SE	LLCI	ULCI
*M* − 1*SD* (1.75)						0.51 ***	0.04	0.42	0.59
*M* (2.47)						0.34 ***	0.03	0.28	0.40
*M* + 1*SD* (3.22)						0.17 ***	0.04	0.09	0.24

Note. *** *p* < 0.001, ** *p* < 0.01.

## Data Availability

The data presented in this study are available on request from the corresponding author.
